# Initial Inoculum and the Severity of COVID-19: A Mathematical Modeling Study of the Dose-Response of SARS-CoV-2 Infections

**DOI:** 10.3390/epidemiologia1010003

**Published:** 2020-10-21

**Authors:** Baylor Fain, Hana M. Dobrovolny

**Affiliations:** Department of Physics & Astronomy, Texas Christian University, Fort Worth, TX 76129, USA; baylor.fain@tcu.edu

**Keywords:** COVID-19, SARS-CoV-2, coronavirus, inoculum, mathematical model

## Abstract

SARS-CoV-2 (Severe acute respiratory syndrome coronavirus 2) causes a variety of responses in those who contract the virus, ranging from asymptomatic infections to acute respiratory failure and death. While there are likely multiple mechanisms triggering severe disease, one potential cause of severe disease is the size of the initial inoculum. For other respiratory diseases, larger initial doses lead to more severe outcomes. We investigate whether there is a similar link for SARS-CoV-2 infections using the combination of an agent-based model (ABM) and a partial differential equation model (PDM). We use the model to examine the viral time course for different sizes of initial inocula, generating dose-response curves for peak viral load, time of viral peak, viral growth rate, infection duration, and area under the viral titer curve. We find that large initial inocula lead to short infections, but with higher viral titer peaks; and that smaller initial inocula lower the viral titer peak, but make the infection last longer.

## 1. Introduction

The novel coronavirus, severe acute respiratory syndrome coronavirus 2 (SARS-CoV-2), first detected in Wuhan, China in late 2019 and rapidly spread around the world [1,2]. While the disease can lead to severe illness needing long hospitalization [3,4,5], a significant fraction of those who contract the virus are asymptomatic [6]. It is still not entirely clear who is at risk for developing severe disease, although correlations of disease severity with levels of vitamin D [7], levels of various immune components [8,9,10,11], and age [10,12] have been noted. There has also been investigation of the possibility of disease severity being linked to initial viral inoculum [13,14,15].

There is some evidence from other respiratory viruses that the size of the initial inoculum could play a role in the severity of the illness. An influenza epidemiological modeling study suggested that a higher initial dose can lead to a higher mortality rate [16]. This is corroborated by an influenza in-host modeling study that also found a correlation between the initial viral dose and survival rate [17]. Other modeling studies have found dependence of other measures of infection severity on initial dose for influenza [18], respiratory syncytial virus [19], adenovirus [20], and porcine reproductive and respiratory virus [21]. There are also experimental studies that find a link between dose and infection severity. Experiments using influenza have found inoculum dose dependence of total number of infected cells and area under the curve [22], peak viral titer [23,24,25], viral growth rate [23], and time of viral peak [23,24]. Experiments with other viruses, such as adenovirus [26] and parainfluenza [27], have also shown correlations between initial inoculum and various measures of disease severity. If SARS-CoV-2 shows a similar pattern, initial inoculum should be considered as a possible contributor to infection severity and adverse outcomes.

The major route of transmission for SARS-CoV-2 is airborne droplets [28]. One study indicates that sneezing and coughing creates a turbulent gas cloud that can cause viral-laden droplets to spread up to 27 feet (7–8m) [29], and allows the virus to get into the ventilation system of a building. A review of literature on droplet and airborne viral spread concludes that 8 of 10 studies showed that droplets spread further than the 6 foot [30] social distancing recommendation. While personal protective equipment is helpful in reducing the ability of virus to enter the respiratory tract, it is not perfect [31]. All of these factors lead to exposures to vastly different quantities of virus when people are going about their daily activities. Thus, it is important to understand whether different initial inocula lead to different viral dynamics in patients.

Given the difficulty of examining SARS-CoV-2 inoculum dependence in patients, our study aims to address the question of inoculum-dependence of SARS-CoV-2 infection severity using mathematical modeling. We use the combination of agent-based model (ABM) and partial differential equation model (PDM) to simulate SARS-CoV-2 infections initiated with different initial inocula. We measure several features of the viral titer curve and find that increasing the initial inoculum leads to an early, high, and narrow peak in the viral titer curve, while decreasing both the infection duration and area under the curve.

## 2. Materials and Methods

### 2.1. Mathematical Model

We use an ABM to model transitions of cells as they go through the infection cycle. We use a hexagonal grid and simulate 10${}^{6}$ cells in a circular dish to mimic an in-vitro system. Cells begin as healthy target cells that can be infected by viruses that are sitting above them. Once infected, the cells move into an eclipse phase where they are not yet actively producing virus. The cells remain in the eclipse phase for a time chosen from an Erlang distribution with mean time $\tau_{E}$ and shape parameter $n_{E}$. The cells then pass into the infectious phase, where they are actively producing virus, for a time chosen from an Erlang distribution with mean time $\tau_{I}$ and shape $n_{I}$, after which time the cells die and no longer participate in the infection. Erlang distributions are used for both transitions based on experiments that show the time spent in the eclipse phase and the time spent in the infectious phase are best described by Erlang distributions [32,33]—at least for SHIV (simian-human immunodeficiency virus). While SHIV is a different virus, it is the only virus for which these distributions have been measured directly. Influenza, another respiratory virus, has also been shown to need nonexponential transition distributions [34,35].

Viral dynamics are described by the PDM as virus diffuses over the layer of cells,

$$\frac{\partial V}{\partial t}=D\nabla^{2}V+p-cV,$$

where *D* is the diffusion coefficient and *c* is the viral decay rate. Virus is produced by infectious cells at rate *p* and is assumed to be released directly above each infected cell. The amount of virus above any cell determines the probability that the cell will be infected, $P_{\inf}=\beta V$, where $P_{\inf}$ is the probability per unit time, and β is the infection rate. A more detailed description of the model is given in the supplementary material, and the simulation code is available on https://github.com/BaylorFain/Covid19-Code.

Parameter values that describe SARS-CoV-2 are taken from a variety of sources and are given in Table 1. The majority of the parameters are taken from [36], where an ordinary differential equation model of coronavirus infection was fit to viral titer data from a single patient. Note that the parameters β and *p* are scaled to account for the different numbers of cells (10${}^{6}$ here and 1 in [36]) in the two systems as well as converting viral concentration to individual virions (see [37,38,39] for detailed discussions on converting from concentration to virions). The shape parameters are based on values derived from influenza infections [40], since the Erlang distribution has not yet been used for SARS-CoV-2. The diffusion coefficient was calculated using the Stokes–Einstein equation [41].

### 2.2. Measurements

**Figure 1:**
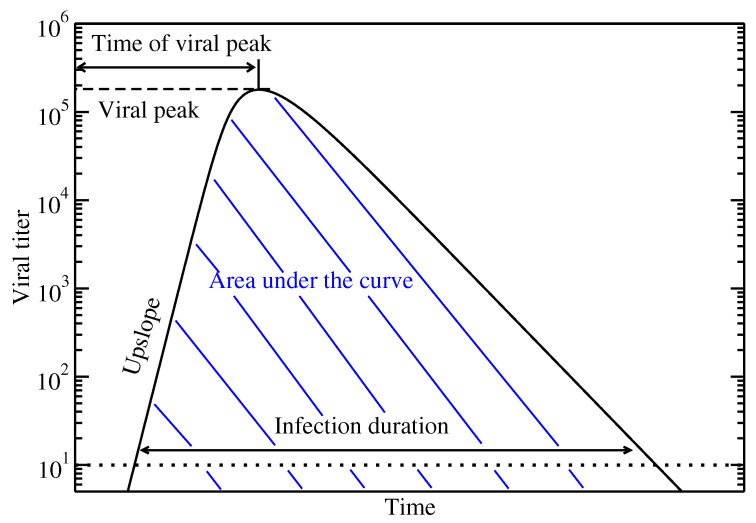
Characteristics of the viral titer curve that are used to assess severity of the infection.

We simulate SARS-CoV-2 infections starting with different multiplicity of infection (MOI), where the MOI value defines the initial number of infected cells. The ABM/PDM model is implemented in Compute Unified Device Architecture (CUDA) and run on NVIDIA graphics processing units. We perform 100 simulated infections for each MOI and measure the following features of the viral titer curve (Figure 1):

- Peak viral load: The maximum amount of virus is commonly used as an indicator of the transmissibility of an infection [42].
- Time of viral peak: This is the time between the start of the infection and the peak of the virus and can give an indication of how quickly the virus is replicating.
- Viral upslope: Viral upslope is the exponential growth rate of the viral titer before the peak is reached and is another indication of how quickly the virus is spreading from cell to cell.
- Area under the curve (AUC): AUC is often used to assess the severity of an infection [43,44].
- Infection duration: The infection duration is indicative of how long an infected patient might test positive for presence of the virus. Note that the threshold used here is 10${}^{7}$ virions based on a 10${}^{2}$ RNA copies/ml detection threshold for the experimental data [45] that is converted to individual virions.

## 3. Results

**Figure 2:**
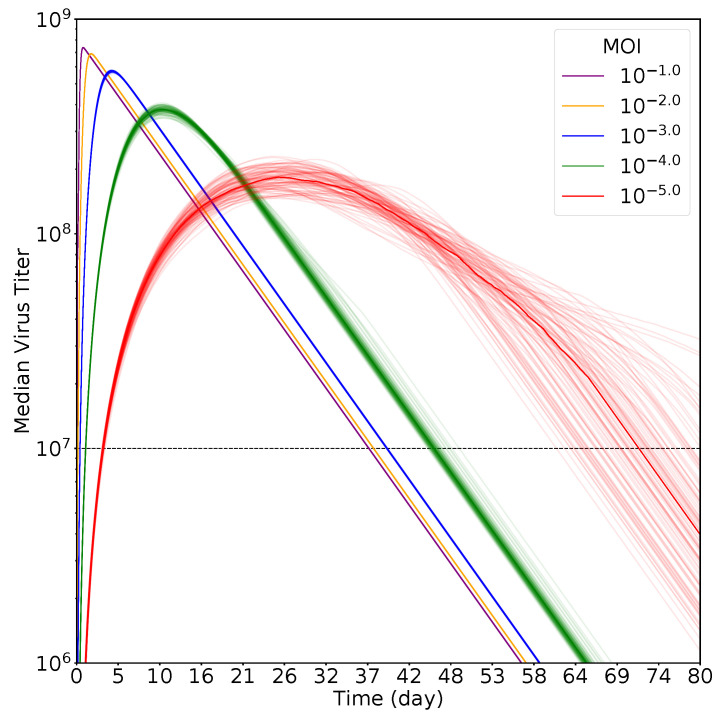
Viral loads for infections initiated with different multiplicity of infection (MOI). Dark lines of each color indicate the viral load curve using the median of 100 simulations, while the lighter colored lines show the viral load kinetics for each individual simulation. The dashed line indicates the threshold of detection used to calculate infection duration.

Figure 2 shows the viral titer curves for different multiplicity of infection (MOI) of SARS-CoV-2, where the darker line for each color shows the curve of median values and the lighter colored lines are the 100 individual simulations. Note that for most MOI, there is very little variation between simulations once the viral titer is large. The exception is the lowest MOI of 10${}^{-5}$ where there is more variation in the exact trajectory of the viral load. We see some obvious shifts in the viral titer curve as the MOI increases. For high MOI, the viral titer curve reaches its peak very quickly, with lower MOIs moving the peak farther out in time. The peak also becomes broader and lower as the MOI becomes lower, suggesting longer infection durations, but with lower viral loads.

**Figure 3:**
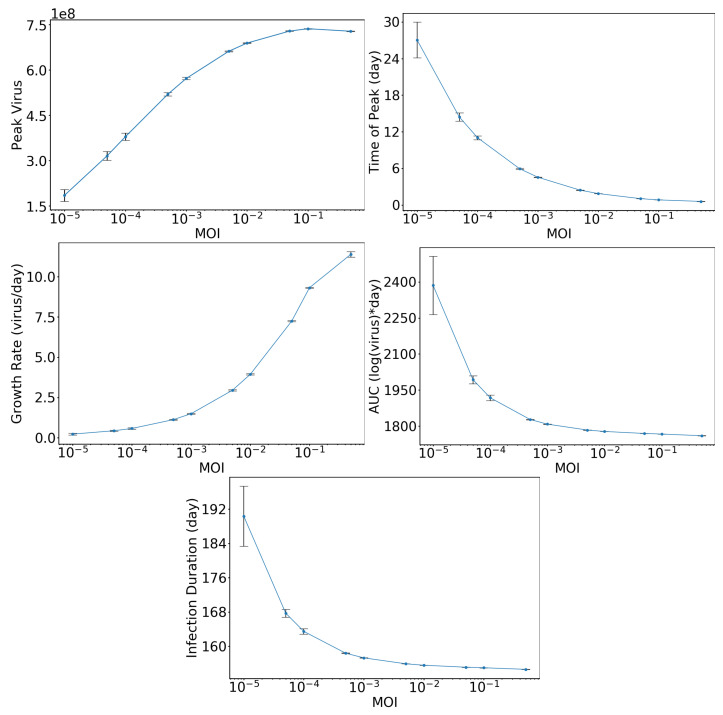
Effect of initial inoculum on viral titer characteristics. The graphs show peak viral load (**top-left**), time of viral peak (**top-right**), viral upslope (**center-left**), area under the curve (AUC) (**center-right**), and infection duration (**bottom**) as functions of MOI.

For a more quantitative assessment, we measure the characteristics described in Methods. The results are shown in Figure 3, which shows peak viral load (top-left), time of viral peak (top-right), viral upslope (center-left), area under the curve (AUC) (center-right), and infection duration (bottom) as functions of the MOI. The peak viral load increases with increasing initial inoculum, but it appears to reach a plateau as we near an MOI of 1. The time of peak, on the other hand, decreases with increasing initial inoculum, reaching a fixed small value at high MOI. There are real plateaus here, since each cell will produce an average of $p\tau_{I}$ viral particles. At an MOI of 1, all cells are initially infected and will start producing virus at about the same time, meaning all of the virus is released almost simultaneously and there is no second cycle of infection. At slightly lower MOIs, most cells are initially infected, but some cells will be infected in a second or third cycle of infection, reducing the large burst of virus at one time, which causes a delay, reduction, and broadening in the peak. The upslope, or growth rate, of the viral titer curve increases as the MOI increases. This is also driven by the larger amount of virus being produced in the first cycle of infection as the MOI increases. Finally, the AUC and infection duration both decrease as the initial inoculum increases.

## 4. Discussion

Our study finds that initial viral inoculum does alter the viral time course by increasing the peak viral load, moving the peak earlier, increasing the viral upslope, and decreasing both AUC and infection duration, as the initial inoculum increases. It is not immediately clear what these changes in viral kinetics mean for the severity of the infection. Is it better to have a shorter infection, albeit with a higher viral peak; or a longer-lasting infection with a lower viral burden? One study compared viral loads in patients with mild and severe illness and found that the viral load time course in mild cases peaked earlier and at a lower peak viral load than in severe cases, although both time courses still had rather high viral loads at 25 days post symptom onset [46]. Since viral load in these patients was measured after they presented at a hospital, there is also no way to link particular features of the viral time course to the initial inoculum. Other observational studies that have attempted to investigate links between viral load and disease severity have taken a limited number of viral load measurements, often well after the peak of the infection [8,47,48], making it impossible to assess the full time course of the viral load and any correlations to initial inoculum. An alternative to observational studies in patients is to investigate inoculum dose-response of SARS-CoV-2 in animals, as suggested in [13]. Such animal studies in conjunction with mathematical modeling studies will help provide a clearer picture of the role of initial inoculum in determining viral time course and disease severity.

We find infection durations ranging from 37–73 days. Studies suggest that median duration of viral shedding is 14–20 days after symptom onset, with some patients shedding virus for more than 30 days after symptom onset [49,50,51,52]. One Italian study found a longer median shedding duration of 36 days after symptom onset [53]. There are, however, cases of patients who have shed virus for longer periods of time, with several case studies finding patients who shed virus for more than 60 days after hospitalization [47,54,55]. In some studies, longer duration of viral shedding is associated with more serious clinical outcomes such as ICU admission or invasive ventilation [52,56], although other studies have noted that asymptomatic patients also seem to shed virus for longer than mildly symptomatic patients [57].

Our findings indicating a decrease in AUC, but an increase in viral peak as MOI increases could be viewed as contradictory since both peak viral load and AUC are supposed to be indicators of disease severity. However, disease severity is often ill-defined. One study has shown a correlation between viral load and total symptom score [58], and another between nasal discharge and viral load [59] for influenza. This implies that a higher peak viral load should lead to higher symptom score, at least around the time of viral peak. Clinical studies, however, tend to use area under the viral curve as an endpoint in studies as an indicator of disease severity [60,61,62,63], perhaps in an attempt to combine both the severity of symptoms and the duration over which symptoms are experienced. This leads back to the question of whether severity should be assessed by the worst period of symptoms, even it is only for a short duration; or whether disease severity should be assessed by milder, but sustained, symptoms.

Viral load on its own is not the only cause of the symptoms experienced by patients. The immune response is thought to underlie many of the symptoms that cause patient discomfort [64] and medical complications [65] for other respiratory viruses. A study using the coronavirus that causes Middle East respiratory syndrome found that high viral load was correlated to high levels of inflammatory cytokines that are, in turn, linked to higher mortality [66]. Several studies have also hypothesized a connection between intensity of the immune response and severe disease for SARS-CoV-2 [67,68,69]. For other respiratory infections, there are several studies that have linked the size of viral inoculum to variations in various components of the immune response [21,70,71,72,73]. Another study links area under neutrophils curve and area under IL-8 curve to symptom severity in respiratory tract infections [74]. Unfortunately, our model does not include an immune response, and so we cannot investigate how immune response might vary with initial inoculum dose and affect the severity of the infection. While mathematical models that include immune responses [75] and symptoms [17,76] have been examined for other respiratory viral infections, there is currently not enough time course data on SARS-CoV-2 immune responses to properly assess the validity of these models for the novel coronavirus.

There are other factors that affect whether a large exposure will lead to severe infection. Simulations show that the site of deposition within the respiratory tract affects not only whether an infection takes hold, but also how easily the virus will replicate [77]. Like other respiratory viruses, SARS-CoV-2 tends to result in more severe infections when it manages to extend to the lower respiratory tract [78]. The ability to spread to the lower respiratory tract seems to be related to mucosal velocity within the respiratory tract [79,80], and not directly to viral replication, so this is yet another factor that needs to be considered in determining the severity of the infection. Since our model does not spatially reproduce the respiratory tract, we also cannot assess how these factors might alter our predictions of viral time course.

The model used here is fairly generic and simulates SARS-CoV-2 only through choice of parameters. However, the effect of initial inoculum on viral titer has not previously been examined in an ABM of viral dynamics. Previous studies using ordinary differential equation (ODE) models suggest that model structure and underlying assumptions change the predicted dose-response [19,20]. Interestingly, the ABM is target-cell limited, and draws its parameter values from a fit of a target-cell limited model to SARS-CoV-2 data, but the dose-response trends observed here are quite different from the dose-response trends observed with a traditional target-cell-limited ODE model [19,20]. For example, in the target-cell-limited ODE, viral titer peak and growth rate do not change with initial inoculum [19,20], but the ABM predicts an increase in both. Time of viral peak and infection duration trends for the ABM are similar to those predicted by target-cell-limited ODEs [19,20].

Despite the limitations of our model, our study found that initial inoculum dose changes the viral time course and that many characteristic features of the viral titer curve change monotonically with the inoculum size. Future studies are needed to extend these results to symptom severity and changes in the immune response to SARS-CoV-2.

## Figures and Tables

**Table 1:** Parameter values to simulate severe acute respiratory syndrome coronavirus 2 (SARS-CoV-2) infection with the agent-based model (ABM)/partial differential equation model (PDM) model.

Parameter	Meaning	Value
$\beta {\;}^{a}$	Infection rate	84.0/h
$\tau_{E}{\;}^{b}$	Mean eclipse duration	5.88h
$n_{E}{\;}^{c}$	Eclipse shape parameter	30
$\tau_{I}{\;}^{b}$	Mean infectious lifespan	0.624h
$n_{I}{\;}^{c}$	Infectious shape parameter	100
$p{\;}^{a}$	Viral production rate	19,900/h
$c{\;}^{b}$	Viral clearance rate	0.00490/h
$D{\;}^{d}$	Diffusion coefficient	$4.80\times 10^{-12}\;m^{2}/s$

${}^{a}$ Parameters taken from [36], but scaled. ${}^{b}$ Parameters taken from [36]. ${}^{c}$ Parameters taken from [40]. ${}^{d}$ Parameter calculated from Stokes–Einstein equation.

## Supplementary Materials

The following are available online at https://www.mdpi.com/2673-3986/1/1/3/s1, more details of the model used in the study are presented in this Supplementary Materials.

## Abbreviations

The following abbreviations are used in this manuscript:

ABM	Agent based model
AUC	Area under the curve
CUDA	Compute unified device architecture
MOI	Multiplicity of infection
ODE	Ordinary differential equation
PDM	Partial differential equation model
SARS-CoV-2	Severe acute respiratory syndrome coronavirus 2
SHIV	Simian-human immunodeficiency virus
