# Supplementary material for: Initial Inoculum and the Severity of COVID-19: A Mathematical Modeling Study of the Dose-Response of SARS-CoV-2 Infections
Source: Epidemiologia (Basel). 2020 Oct 21;1(1):5–15. doi: 10.3390/epidemiologia1010003 (PMC9620883; doi:10.3390/epidemiologia1010003)
Supplement: Supplementary file 1 [file epidemiologia-01-00003-s001.pdf]

Supplement for "Initial inoculum and the severity of COVID-19: A mathematical modeling study of the dose-response of SARS-CoV-2 infections"

Baylor Fain and Hana M. Dobrovolny

In this supplement we present more details of the model used in the study.

1 Model details

An ABM breaks a system down into smaller units, or agents, and each agent is governed by a set of rules. As the simulation is stepped through time, the agents act and interact. These actions cause bulk properties, at the system level of the simulation, to become apparent for observation and measurement. In any culture dish there is on the order of 10^6 cells [5], so we simulate 10^6 agents — this will allow for the simulation to reproduce what is happening in the dish. The dish in which the cells are grown will be assumed to have been grown to confluence. The best representation for confluence is for the cells to be represented as hexagons on a hexagonal spatial grid, because the cells are squashed in by their nearest 6 neighbors.

In order for the hexagonal grid to be incorporated into the ABM, there must be a way to identify and index through each of the hexagons. There are different conventions that can be followed to make a grid of hexagons; one way is cube coordinates. In cube coordinates there are three numbers X_{hex}, Y_{hex}, and Z_{hex} that are used to identify a hexagon. Often people are familiar with cube coordinates if they want to plot hexagons on an xy plot, because the conversion to 2-D cartesian coordinates from hexagonal coordinates is straightforward:

$$x = X_{hex}$$

and

$$y = 2/3 \sin(\pi/3) \left(Y_{hex} - Z_{hex}\right).$$

There are three other attributes to cube coordinates that are useful for making hexagonal grids:

1. The coordinates can be split in to three sectors where the coordinates X_{hex}, Y_{hex}, and Z_{hex} are simply cyclic permutations.

2. The X_{hex} and Z_{hex} coordinates, also known as axial coordinates, can be used as indices of a matrix.

3. The coordinates of the neighboring hexagons are found by adding a cyclic permutation of $\begin{bmatrix} 1 \\ 0 \\ -1 \end{bmatrix}$ for three of the neighbors and $\begin{bmatrix} 1 \\ -1 \\ 0 \end{bmatrix}$ for the other three neighbors.

Attribute 1 allows for quick construction of grids, by reducing the number of hexagon locations to be calculated by a third. Attributes 2 and 3 give the ability to store the hexagon locations in a matrix allowing for simple referencing of the hexagons and their neighbors in a computer code. We assume that each hexagon is a single cell.

The ABM governs the transitions a cell makes through the stages of infection: healthy, eclipse, infected, and dead. A cell in the healthy state is an uninfected cell that remains healthy until infected, either through cell-free transmission or cell-to-cell transmission. A cell in the eclipse state is an infected cell that is not yet producing virus. The cell will remain in the eclipse state until it transitions to the infected state. A cell in the infected produces virus and will continue in this state until it dies. A cell in the dead state is a cell that can no longer change state, therefore once a cell is in the dead state the cell will remain in that state until the end of the simulation. The flow of this is illustrated in figure 1.

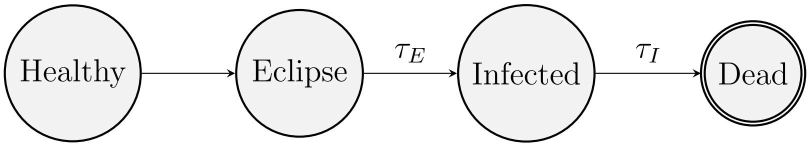

Figure 1: The stages of infection healthy, eclipse, infected, and dead are shown. Cells stay in the eclipse stage for an average time τ_E and in the infected stage for an average time τ_I.

To apply the ABM computationally, four arrays with an element for each cell are created: universal time (UT), healthy time (HT), eclipse time (ET), and infected time (IT). The universal time array holds the amount of time that each cell has been in the simulation, each element starts at zero and increases each iteration by the simulation's time step. The healthy time array holds the amount of time that a cell is healthy, each element for a healthy cell starts at zero and increases each iteration by the simulation's time step. The eclipse time array holds the amount of time each cell will be in the eclipse state. Each element in the array is initially filled with a different number pulled from a gamma distribution with shape value τ_E and scale value $\tau_E/\sqrt{\eta_E}$. We use gamma distributions for these transitions since recent experimental work suggests that these transitions follow gamma (Erlang) distributions [2, 4]. The infected time array holds the amount of time each cell will be in the infected state. Each element in the array is initially filled with a different number pulled from the gamma distribution with shape value τ_I and scale value $\tau_I/\sqrt{\eta_I}$. Through tracking these "time" arrays the ABM can transition cells to different states after infection.

In the culture dish, as an infected cell releases virus into the extracellular fluid, the virus diffuses across a density gradient. With the PDM, diffusion is represented by the diffusion equation,

$$\frac{\partial V}{\partial t} = D\nabla^2 V + p - cV, \tag{1}$$

where V is the concentration of the virus, D the diffusion coefficient, p is the production rate per infected cell, and c is the clearance rate of the virus. Since we are using a hexagonal grid, this becomes,

$$\frac{\partial V}{\partial t} = D\frac{2}{3}\left(\frac{\partial^2}{\partial x_1^2} + \frac{\partial^2}{\partial x_2^2} + \frac{\partial^2}{\partial x_3^2}\right)V + p - cV, \tag{2}$$

where x_i are the unit vectors for a hexagonal grid, $\mathbf{x}_1 = \begin{bmatrix} 1 \\ 0 \end{bmatrix}$, $\mathbf{x}_2 = \begin{bmatrix} -1/2 \\ \sqrt{3}/2 \end{bmatrix}$, $\mathbf{x}_3 = \begin{bmatrix} -1/2 \\ -\sqrt{3}/2 \end{bmatrix}$. In the code, along with the assumption of hexagonal cells, the cells will be assumed to be flat, so the virus is diffusing over a smooth 2-D plane.

We assume cells are infected via cell-free transmission where cells must leave one cell and diffuse over the surface of the cell layer before infecting other cells [1]. In the model, the probability (P) that a cell will become infected is defined by the amount of virus that is covering the cell [3],

$$P = \beta V \Delta t.$$

As a healthy cell becomes covered in virus the probability of cell free infection increases. If the probability is ever greater than one due to the build up of virus, an adaptive time step is used. The time step is divided in half repeatedly, until the probability is below one. Once the probability is finalized, a number is pulled from the uniform distribution and compared with the probability. If that number is less than the probability, then the cell will become infected. The ABM/PDEM model is implemented in Compute Unified Device Architecture (CUDA) and run on NVIDIA graphics processing units.

References

[1] L. J. Allen and E. J. Schwartz. Free-virus and cell-to-cell transmission in models of equine infectious anemia virus infection. *Math. Biosci.*, 270:237–248, December 2015. doi: 10.1016/j.mbs.2015.04.001.

[2] C. A. Beauchemin, T. Miura, and S. Iwami. Duration of SHIV production by infected cells is not exponentially distributed: Implications for estimates of infection parameters and antiviral efficacy. *Sci. Rep.*, 7:42765, 16 February 2017. doi: 10.1038/srep42765.

[3] B. P. Holder, L. E. Liao, P. Simon, G. Boivin, and C. A. A. Beauchemin. Design considerations in building in silico equivalents of common experimental influenza virus assays and the benefits of such an approach. *Autoimmunity*, 44(4), June 2011. doi: 10.3109/08916934.2011.523267.

[4] Y. Kakizoe, S. Nakaoka, C. A. Beauchemin, S. Morita, H. Mori, T. Igarashi, K. Aihara, T. Miura, and S. Iwami. A method to determine the duration of the eclipse phase for in vitro infection with a highly pathogenic SHIV strain. *Sci. Rep.*, 5:10371, 21 May 2015. doi: 10.1038/srep10371.

[5] L. T. Pinilla, B. P. Holder, Y. Abed, G. Boivin, and C. A. A. Beauchemin. The H275Y neuraminidase mutation of the pandemic A/H1N1 influenza virus lengthens the eclipse phase and reduces viral output of infected cells, potentially compromising fitness in ferrets. *J. Virol.*, 86(19):10651–10660, October 2012. doi: 10.1128/JVI.07244-11.
